# Prevalence of Hepatitis D Virus Infection Among Hepatitis B Virus Infected Patients in Qom Province, Center of Iran

**DOI:** 10.5812/hepatmon.847

**Published:** 2012-03-28

**Authors:** Mohammad-Reza Ghadir, Mojtaba Belbasi, Akram Heidari, Seyed Saeid Sarkeshikian, Alireza Kabiri, Amir Hossein Ghanooni, Abolfazl Iranikhah, Maryam Vaez-Javadi, Seyed Moayed Alavian

**Affiliations:** 1 Gastroenterology Section, Department of Internal Medicine, Faculty of Medicine, Qom University of Medical Sciences, Qom, IR Iran; 2 Iranian Blood Transfusion Organization Research Center, Qom, IR Iran; 3 Faculties of Medicine, Qom University of Medical Sciences, Qom, IR Iran; 4 Qom University of Medical Sciences, Qom, IR Iran; 5 Gastroenterology Section, Department of Pediatrics, Faculty of Medicine, Qom University of Medical Sciences, Qom, IR Iran; 6 Baqiyatallah Research Center for Gastroenterology and Liver Diseases, Baqiyatallah University of Medical Sciences, Tehran, IR Iran

**Keywords:** Hepatitis D, Hepatitis B Virus, Prevalence, Iran

## Abstract

**Background:**

Hepatitis D virus (HDV) is a defective RNA virus that depends on the hepatitis B surface antigen (HBsAg) of hepatitis B virus for its replication, developing exclusively in patients with acute or chronic hepatitis B. There are little data regarding the routes of HDV transmission in Iran. The risk factors for HDV infection in Iran are blood transfusion, surgery, family history, Hejamat wet cupping (traditional phlebotomy), tattooing, war injury, dental interventions, and endoscopy.

**Objectives:**

We performed this study to determine the prevalence of hepatitis D in the general population of Qom province and the potential risk factors for acquiring HDV.

**Patients and Methods:**

This cross-sectional study collected 3690 samples from 7 rural clusters and 116 urban clusters. HBs antigen was measured, and if the test was positive, anti-HDV was measured. Ten teams, each consisting of 2 trained members, were assigned to conduct the sampling and administer the questionnaires. The data were analyzed using SPSS.

**Results:**

Forty-eight subjects (1.3%) suffered from hepatitis B, and 1 HBsAg-positive case had HDV infection. The prevalence of hepatitis D infection in Qom Province was 0.03%. The prevalence of hepatitis D infection in HBsAg-positive cases was 2%. Our anti-HDV-positive case had a history of tattooing, surgery, and dental surgery. There was no significant relationship between tattooing, surgery history, or dental surgery and hepatitis D infection.

**Conclusions:**

The prevalence of hepatitis D in Qom is the the lowest in Iran, similar to a study in Babol (north of Iran).

## 1. Background

Hepatitis D virus (HDV) is a defective RNA virus that depends on the hepatitis B surface antigen (HBsAg) of hepatitis B virus for its replication, developing exclusively in patients with acute or chronic hepatitis B. Simultaneous infection with HDV tends to accelerate the progression of chronic hepatitis B virus (HBV) to chronic active hepatitis, cirrhosis, and hepatocellular carcinoma and mediates fulminant hepatitis. In addition, the response of HDV patients to antiviral therapy and the required dosages of therapeutic regimens differ from those of chronic hepatitis B alone [1][2]. Approximately 5% of patients with chronic hepatitis B infection worldwide are infected with hepatitis D virus. Its prevalence in Italy, eastern Europe, and western Asia is higher than in the rest of the world, reaching 83.3%, 8.3%, and 12.5% in Romania, Italy, and Russia, respectively [2][3][4]. Hepatitis D virus (HDV) has a broad geographical distribution, with 2 dominant patterns of transmission. In endemic regions, such as southern Italy, parts of Africa, and South America, it is transferred through close personal contact in the absence of clear skin contact, such as close personal relationships among members of a family. In contrast, in areas that have a low prevalence, such as western Europe and North America, HDV is seen more commonly in groups with frequent skin contact, such as continual recipients of blood and blood products and intravenous drug users [5][6]. Sexual transmission and maternal-child transmission are other modes [1][7]. There are little data regarding the routes of HDV transmission in Iran. The predominant routes of the transmission of HBV in Iran are maternal, from infected mothers to infants, and horizontally during childhood [8]. The epidemiology of hepatitis B has shifted in Iran, and horizontal transmission in adults is increasing [9]. The risk factors for HDV infection in some studies in Iran have been blood transfusion, surgery, family history, wet cupping Hejamat (traditional phlebotomy) ,tattooing, war injury, dental interventions, and endoscopy [5][6]. The most frequent method of diagnosing HDV infection is the measurement of anti-HDV (IgM, IgG) in serum by ELISA. PCR can also be used to detect viral RNA in blood [10][11]. Acute HDV infection can occur simultaneously with acute HBV infection or can be superimposed onto chronic HBV infection. Fatal fulminant hepatitis occurs in 20% to 30% of coinfections of HDV and HBV in humans versus 2% of patients with acute hepatitis B without HDV coinfection [7].

## 2. Objectives

We performed this study to determine the prevalence of hepatitis D in the general population of Qom province (center of Iran) and the potential risk factors for acquiring HDV.

## 3. Patients and Methods

This study was a cross-sectional descriptive study. A population of 3960 individuals was assigned to the study by cluster sampling. Seven rural clusters and 116 urban clusters were established, based on girls' elementary schools in different districts of the province. The clusters were selected according to the proportion of urban and rural populations in Qom. From each cluster, 30 blood specimens were obtained. For sampling purposes, only 1 person from each family was selected randomly by draw of the lots. Then, the phases of the study were explained to the individuals who were eligible for the study. Those who were interested were invited to undergo tests and complete questionnaires. The inclusion criteria were HbsAg positivity, and those who were not Iranian were excluded. After obtaining written consent from the candidates, a questionnaire, including such data as age; sex; marital status; and history of blood transfusion, surgery, dental surgery, viral hepatitis, immunization, narcotic drug abuse, smoking, tattooing, cupping, and endoscopy, was completed for each candidate. Nearly 5 cc of serum was taken from each subject and transferred to the lab under sterile conditions in ice bags. HBs antigen was measured at the lab, and if the test was positive, anti-HDV was measured by ELISA (Biovendor, Germany). Ten teams of 2 trained members were responsible for preparing the samples and completing the questionnaires. Categorical variables were analyzed by Pearson chi-square test and Fisher exact test. The data were analyzed using SPSS, and P < 0.05 was considered significant.

## 4. Results

This study determined the prevalence of hepatitis D in Qom Province. Only 1 patient was infected with hepatitis D. Of the 3690 subjects, 48 (1.3%) suffered from hepatitis B. Fifty-six percent of HBsAg-positive cases were male (Table 1). Thus, 2% of infected patients and 0.03% of the entire group had HDV infection. Our anti-HDV-positive case was a married 31-year-old woman who lived in Qom who originated from Afghanistan and had a history of tattooing, surgery, and dental surgery. By Fisher test, there was a significant relationship between tattooing and hepatitis D infection in the entire population (P = 0.045), but there was no significant relationship between surgical history or dental surgical history and hepatitis D in the entire group (P > 0.05). Of cases with an HBsAg-positive test, there was no significant relationship between tattooing, surgical history, or dental surgical history and hepatitis D infection (P > 0.0586).

**Table 1:** Seoprevalence of HBs Antigen and HDV Antibody According to Demographic Characteristics in Qom, Iran

	**HBs-Ag Positive, No. (%)**	**HDV-Ab Positive, No. (%)**	**P value**
Gender			> 0.05
Male	27 (56)	0 (0.00)	
Female	21 (44)	1 (4.7)	
Drug Addiction			> 0.05
Yes	0 (0.00)	0 (0.00)	
Occasionally	2 (4.34)	0 (0.00)	
No	44 (95.6)	1 (2.27)	
Smoking			> 0.05
Yes	7 (14.58)	0 (0.00)	
No	41 (85.41)	1 (2.43)	
Tattooing			> 0.05^a^
Yes	6 (12.5)	1 (16.66)	
No	42 (87.5)	0 (0.00)	
Marital Status			> 0.05
Single	6 (13.63)	0 (0.00)	
Married	38 (86.36)	1 (98.61)	
Transfusion History			> 0.05
Yes	2 (4.25)	0 (0.00)	
No	45 (95.74)	1 (2.22)	
Dialysis History			> 0.05
Yes	0 (0.00)	0 (0.00)	
No	47 (1.00)	1 (2.12)	
Surgery History			> 0.05
Yes	1 (2.17)	1 (100.00)	
No	45 (97.82)	0 (0.00)	
Cupping History			> 0.05
Yes	6 (12.5)	0 (0.00)	
No	42 (87.5)	1 (2.38)	
Dental Procedure History			>0.05
Yes	29 (60.41)	1 (3.44)	
No	19 (39.58)	0 (0.00)	
Endoscopic History			> 0.05
Yes	7 (14.58)	0 (0.00)	
No	41 (85.41)	1 (2.43)

^a^ P value in whole subjects = 0.045

## 5. Discussion

There are data from different parts of the country reporting disparate prevalence rates. In a study by Malekzadeh et al. in asymptomatic hepatitis B carriers in Shiraz (South of Iran), 13.9% was positive for anti-HDV [12]. This was the first report on the epidemiology of HDV infections from Iran. In a new study by Taghavi et al. in Shiraz in chronic hepatitis B patients over the age of 15 years old, the anti-HDV positivity rate was 9.7%, demonstrating a decrease in prevalence [13]. Amini et al. reported that 2.4% of HBsAg carriers in Hamadan (midwest of Iran) were positive for anti-HDV-a low prevalence of HDV infection. Positive HDV cases were mainly children and young adults (< 20 years of age), suggesting that superinfection or coinfection with HDV occurred in childhood or early adolescence [14]. In a study in Tehran, Rezvan et al. reported that 2.4% of HBsAg-positive blood donors and 44.5% of hemodialysis patients with HBsAg had anti-HDV [15]. Karimi et al. reported that 1.3% of HBsAg-positive blood donors and 25.2% of hemodialysis patients with HBsAg were positive for anti-HDV in Tehran [16]. Alavian et al . reported that 5.7% of chronic hepatitis B patients in Tehran (capital of Iran) were anti-HDV-positive. A history of transfusion, surgical history, tattooing, war injury, dental interventions, and endoscopy were common in HDV infection and were the risk factors [5]ċC. Roshandel et al. reported that 5.8% of HBsAg-positive individuals had anti-HDV in Golestan [17][18][19]. Hasanjani Roushan et al. reported that 2% of chronic hepatitis B patients were anti-HDV-positive in Babol (north of Iran) [20], whereas Zahedi et al. reported that 20.7% of chronic hepatitis B patients were anti-HDV-positive in Kerman (south of Iran) [21]. Torabi et al. reported a prevalence of 6.15% for anti-HDV-positivity in chronic hepatitis B patients in Tabriz (northwest of Iran) [22]. Vaziri et al. noted a prevalence of 31.57% for anti-HDV positivity in HIV patients who were coinfected with HBV in Kermanshah (west of Iran) [23]. Ataei et al. reported that there was no association between hepatitis D and probable risk factors [24]. In conclusion, HDV infection is a widespread disease that has affected a large number of the HBV-infected population in Iran and is considered to be a major public health problem in Iran. The heterogeneous geographic distribution of HDV infection throughout the country indicates that the risk factors of HDV infection differ between regions of the country and that comprehensive surveys in HBV-infected patients should be conducted to determine the risk factors and prevalence of infection [25]. This study showed that the prevalence of HDV in Qom province is low versus other provinces in Iran-the Roushan study in Babol. The lowest rate of HDV was in Qom (center of Iran) and Babol (north of Iran). Although our positive case had a history of tattooing, surgery, and dental surgery, the only significant relationship in the entire population was between HDV infection and tattooing (P = 0.045); there was no significant relationship between the other risk factors and HDV infection in the entire population or in the HBsAg-positive cases (P > 0.05).
